# A Cryptic Targeting Signal Creates a Mitochondrial FEN1 Isoform with Tailed R-Loop Binding Properties

**DOI:** 10.1371/journal.pone.0062340

**Published:** 2013-05-13

**Authors:** Lawrence Kazak, Aurelio Reyes, Jiuya He, Stuart R. Wood, Gloria Brea-Calvo, Torgeir T. Holen, Ian J. Holt

**Affiliations:** 1 MRC-Mitochondrial Biology Unit, Wellcome Trust-MRC Building, Cambridge, United Kingdom; 2 Institute of Basic Medical Sciences, Oslo, Norway; 3 MRC-National Institute for Medical Research, Mill Hill, London, United Kingdom; Keio University, Japan

## Abstract

A growing number of DNA transacting proteins is found in the nucleus and in mitochondria, including the DNA repair and replication protein Flap endonuclease 1, FEN1. Here we show a truncated FEN1 isoform is generated by alternative translation initiation, exposing a mitochondrial targeting signal. The shortened form of FEN1, which we term FENMIT, localizes to mitochondria, based on import into isolated organelles, immunocytochemistry and subcellular fractionation. In vitro FENMIT binds to flap structures containing a 5′ RNA flap, and prefers such substrates to single-stranded RNA. FENMIT can also bind to R-loops, and to a lesser extent to D-loops. Exposing human cells to ethidium bromide results in the generation of RNA/DNA hybrids near the origin of mitochondrial DNA replication. FENMIT is recruited to the DNA under these conditions, and is released by RNase treatment. Moreover, high levels of recombinant FENMIT expression inhibit mtDNA replication, following ethidium bromide treatment. These findings suggest FENMIT interacts with RNA/DNA hybrids in mitochondrial DNA, such as those found at the origin of replication.

## Introduction

Nuclear genes encode the vast majority of mitochondrial proteins [1] which are synthesized by ribosomes in the cytosol, and subsequently imported into the organelle [2]. The import of proteins into the mitochondrial matrix is frequently dependent on an amino (N)-terminal, positively charged amphipathic α helix, which functions as a mitochondrial targeting signal (MTS). Among the many nuclear-encoded mitochondrial gene products, are factors dedicated to the maintenance and expression of mitochondrial DNA (mtDNA) [3], [4]. Other factors contributing to mtDNA metabolism belong to the burgeoning group of proteins that are targeted to multiple cellular compartments. In some cases, a single protein is dually localized [5], more often, multiple protein isoforms are synthesized from a single gene, via the use of alternative splice sites or transcription start sites, or from a single transcript by means of alternative translation initiation (ATI) sites [6].

ATI, first discovered in viruses [7], [8], is one of the gene regulatory mechanisms that diversifies the mammalian proteome. The generation of N-terminal protein variants by ATI may alter a protein's function or cellular location. If initiation from the first AUG includes an MTS then the product will be directed to mitochondria, whereas the use of an internal start site creates a form of the protein destined for other compartments of the cell [9]–[11]. Moreover, because the MTS is typically removed following mitochondrial import, essentially the same protein can be made for two compartments from a single transcript. However, the ability of ATI to expose a cryptic MTS, located within the coding sequence of a protein, is less well documented. Products generated in this fashion would create N-terminally truncated mitochondrial isoforms.

Flap endonuclease 1 (FEN1) has been implicated in processing nucleic acid intermediates formed during lagging strand DNA replication and DNA repair in the nucleus [12]–[15]. Fen1 deletion in the mouse results in embryonic lethality [16], and mutations in the gene give rise to human cancer [17]. While the majority of FEN1 localizes to nuclei [18], it has also been detected in mitochondria [19], [20], where it has been linked to long-patch base excision repair (LP-BER) [20]. However, the same role has been ascribed to exo/endonuclease EXOG [21], although the phenotype associated with EXOG knockdown may be dependent on replication, as opposed to repair defects, as the effects on mtDNA were observed in the absence of any external DNA damaging agent. One of the aforementioned studies of FEN1 detected another polypeptide in mitochondria, cross-reacting with a FEN1 antibody [20]. This protein was shorter than FEN1 and was associated with mitochondrial nucleoids.

Here we show that translation from an internal start codon creates a mitochondrial isoform of FEN1. Its existence and location is confirmed by subcellular fractionation, immunocytochemistry and mitochondrial import. In vitro, the preferred substrates of the truncated form of FEN1 are R-loops. In the major non-coding region of mtDNA, where replication frequently starts, are GC-rich sequences that promote R-loop formation [22]. Hence, truncated FEN1 potentially has a role in stabilizing such structures, and consistent with this hypothesis the protein is recruited to mtDNA when RNA/DNA hybrids accumulate near the origin of replication.

## Results

### Alternative translation initiation creates a mitochondrial specific FEN1 isoform

**Figure 1:**
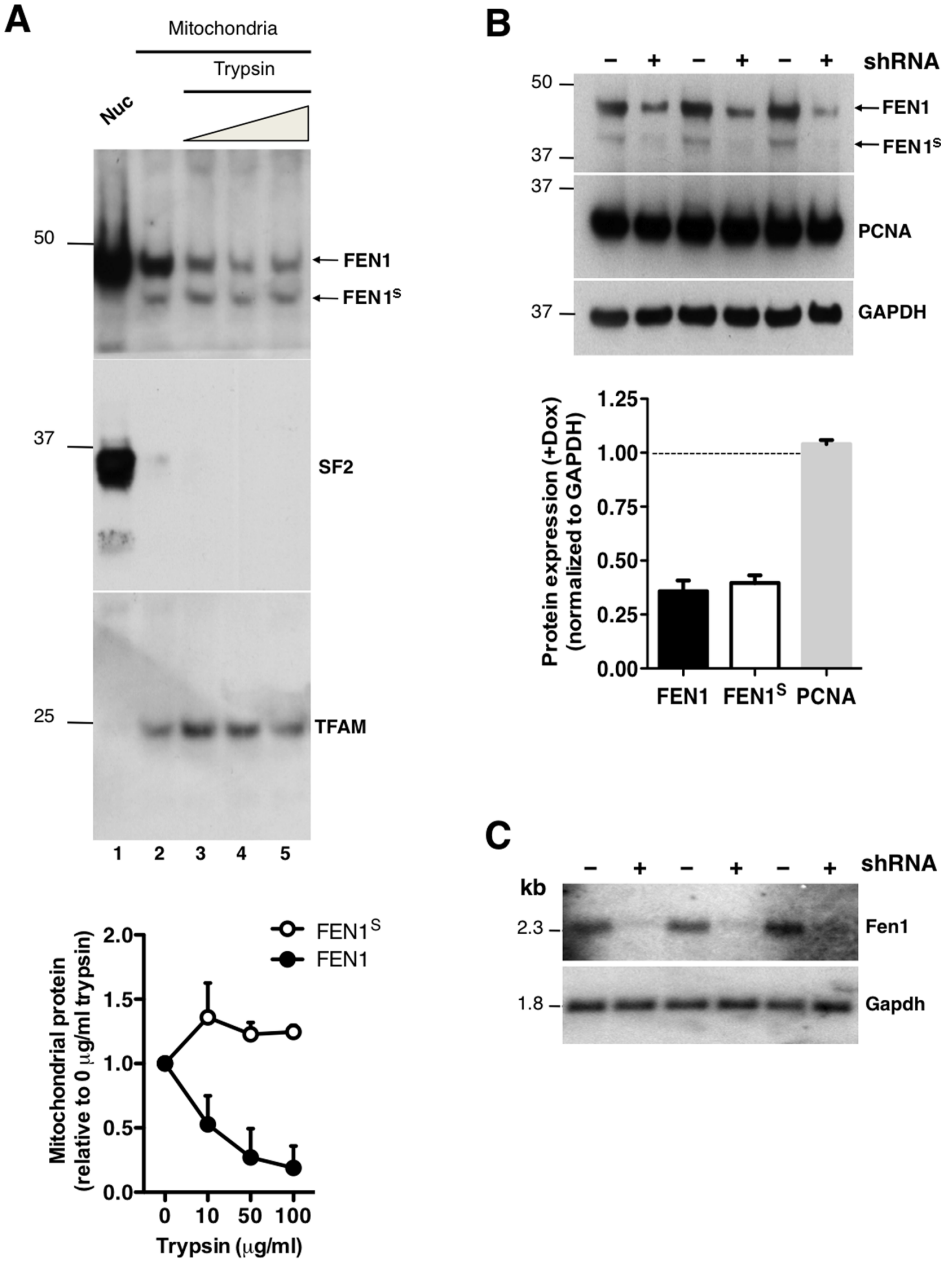
FEN1 antibody detects two polypeptides in mitochondria that are depleted by shRNA targeting the FEN1 transcript. (**A**) Nuclei (Nuc - lane 1) and trypsin-treated mitochondria were subjected to immunoblotting for endogenous FEN1. Trypsin concentrations were: 0, 10, 50 and 100 µg/ml (lanes 2–5, respectively). SF2 (Splicing factor 2) and TFAM were used as nuclear and mitochondrial markers, respectively. FEN1 and FEN1^S^ signal was quantified by densitometry, and the signal of mitochondrial FEN1 (black circles) and FEN1^S^ (white circles) was expressed relative to 0 µg/ml trypsin (lane 2), normalized to 1: see the chart below the immunoblots. Error bars represent standard error of the mean of three independent experiments. FEN1^S^ was also detected with another commercial FEN1 antibody (Bethyl) (data not shown). (**B**) Total protein was extracted from Fen-Rex cells [48] following treatment without (-) or with (+) doxycycline (1 ng/ml) to express an shRNA targeted against the Fen1 transcript. PCNA and GAPDH were used as loading controls. Quantification of immunoblots was done by densitometry, and is shown below the SDS-PAGE image. (**C**) After shRNA induction, as per panel B, total RNA was extracted from Fen-Rex cells and analyzed by Northern blotting using probes targeting Fen1 and Gapdh mRNA. For oligonucleotide sequences, see Text S1.

FEN1 has well defined roles in DNA replication and repair in the nucleus and it has also been detected in mitochondria, where its functions are less well understood [12], [18], [20]. A protein shorter than the annotated FEN1 was detected with an antibody to FEN1 in HeLa cells and shown to associate with mtDNA [20]. Using antibodies to FEN1, we detected two endogenous proteins in isolated mitochondria from one primary, and four immortal human cell lines (Figure 1A, S1A, S1B), the shorter of which (FEN1S) was enriched in mitochondria (Figure 1A). Limited protease treatment of intact mitochondria is a key assay for assigning proteins to internal compartments of these organelles, as it can be used to degrade proteins outside mitochondria and constituents of the outer mitochondrial membrane [23]. Although the endogenous FEN1S appeared less abundant than the full-length protein in mitochondrial preparations (Figure 1A, lane 2), they were similar in amount after removal of non-mitochondrial FEN1 with trypsin (Figure 1A, compare lane 2 to lanes 3–5). Quantification of the relative amounts of FEN1 and FEN1S showed the majority (∼75%) of FEN1 that associated with isolated mitochondria was sensitive to protease treatment, i.e.∼75% of FEN1 co-purifying with the organelles is located outside mitochondria (Figure 1A chart). In contrast to FEN1, FEN1S signal increased by∼25% after trypsin treatment (Figure 1A chart). Furthermore, after mitochondrial sub-fractionation on Iodixanol gradients [24], FEN1S appeared to be the more abundant of the two proteins (Figure S1A, S1B). However, little if any FEN1S co-fractionated with mtDNA, with the majority resolving close to the top of the gradient, where soluble proteins are found. Thus, the majority of the putative short form of FEN1 is not tightly bound to mtDNA after mitochondrial lysis with n-Dodecyl β-D-maltoside. Expression of a short hairpin RNA targeting Fen1 mRNA confirmed that the two species detected by the antibody are products of the FEN1 gene, as both were repressed to similar extents (Figure 1B), and Northern blotting suggested that they derive from a single transcript (Figure 1C). Therefore, we examined the sequence of FEN1 for potential internal translation start sites that could expose a MTS [23]. According to several mitochondrial prediction programs, a hypothetical FEN1 protein starting at methionine 65 (M65) in human and mouse will be targeted to mitochondria (Figure S1C), suggesting that ATI could generate a mitochondrial isoform of mammalian FEN1.

When full-length Fen1 cDNA, preceded by a consensus Kozak sequence [25], was introduced into a coupled transcription/translation (TnT) system, translation products initiating from M1, M37 and M65 (or M67) were detected, based on comparisons with truncated templates (Figure S2A, lanes 1–4). M1 was the most, and M65 the least, abundant product, suggesting that initiation at M65 occurs according to a leaky ribosomal scanning mechanism [26]. An appropriate Kozak setting appears to be another relevant parameter for translation initiation of FEN1, as replacing the artificial consensus Kozak sequence with 21 nucleotides of the native wild type Fen1 mRNA upstream of the AUG encoding M1 (WT M1), reduced translation from M1, and increased translation from M65 (Figure S2A, compare lanes 4 to 5 and see chart). However, the AUG encoding M37 is flanked by nucleotides that more closely resemble an optimal Kozak sequence, than those of M65 (Figure S2B), and so this model could not explain why the increase in initiation from M65 was greater than that from M37. The bias towards M65 might depend on a pair of out-of-frame small ORFs (sORFs) terminating at M37 of FEN1 (Figure S2C). If utilized by ribosomes, these sORFs will limit initiation events from M37, in the manner proposed for RNase H1 [10]. Consistent with this supposition, ablation of the two sORFs led to a significant increase in initiation from M37, in vitro (Figure S2D). Thus, three structural features of the FEN1 mRNA are implicated in the ATI mechanism: the Kozak sequences flanking the AUGs, the presence of two sORFs within the mRNA and the 5′ untranslated region immediately adjacent to the first initiator codon (M1).

Many proteins destined for the mitochondrial compartment of the cell, such as transcription factor A of mitochondria (TFAM), can be imported into isolated organelles. To determine if this was the case for one or more of the FEN1 variants, the labeled TnT products were incubated with isolated rat liver mitochondria. The predicted dATI isoform starting at M65, FEN165, was imported in a membrane potential-dependent manner, with an efficiency of∼40% relative to TFAM, at least five times that of the other FEN1 variants (Figure S2E). Removing an additional two amino acids (FEN167) resulted in an eight-fold decrease in import efficiency, and there was essentially no import of FEN137 (0.5% of TFAM import). Although, FEN11 was imported inefficiently relative to TFAM (8%), this was 16 fold higher than FEN137 and so was well above background. Moreover, inefficient mitochondrial import is fully compatible with the protein's known major destination, the nucleus; this finding supports the contention that mitochondria contain some full-length FEN1 [19], [20].

**Figure 2:**
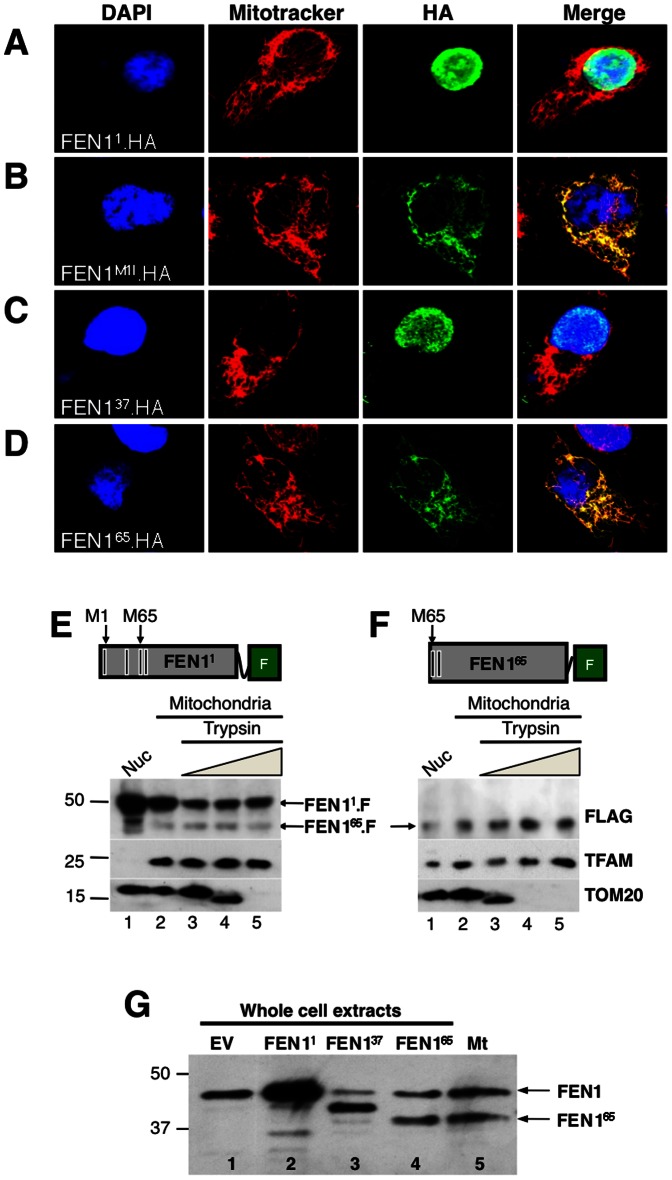
The M65 dATI variant of human FEN1 is targeted to mitochondria. Immunocytochemistry of Human Osteosarcoma (HOS) cells transiently transfected with human FEN1 constructs with HA-tags on the C-terminus to determine cellular localization. (**A**) Full-length FEN1 (FEN1^1^.HA), (**B**) M1 mutated to isoleucine (FEN1^M1I^.HA), (**C**) truncated FEN1, equivalent to dATI from M37 (FEN1^37^.HA), and (**D**) truncated FEN1, equivalent to dATI from M65 (FEN1^65^.HA) were used. Recombinant proteins were labeled with anti-HA antibody (green), while nuclei (blue) and mitochondria (red) were visualized by staining cells with DAPI and Mitotracker, respectively. Purified nuclei and mitochondria were isolated from Flp-In™ T-Rex™ 293 (HEK293T) cells expressing (**E**) FEN1^1^ or (**F**) FEN1^65^ transgenes, tagged with Flag (F) on their C-termini (FEN1^1^.F and FEN1^65^.F, respectively). Mitochondria were subjected to trypsin protection assays, followed by immunoblotting. Transgenes were expressed by the addition of 10 ng/ml doxycycline (Sigma) for 24 h. Schematic representations of the transgenes are depicted above the immunoblots. Nuclei (Nuc. - lane 1); beige slope, increasing trypsin concentrations of 0, 10, 50 and 100 µg/ml (lanes 2–5, respectively). Ectopic FEN1 was detected with anti-FLAG. Mitochondrial markers were TFAM and TOM20 (translocase of the outer membrane 20). E and F are from a single gel, and a similar experiment can be seen in Figure S3B. The band in lane 1 located below full-length FEN1, but higher than FEN1^65^, is probably a degradation product of full-length FEN1 due to excess of recombinant protein. (**G**) Anti-FEN1 immunoblot of whole cell protein extracts from HOS cells overexpressing empty vector (EV) (lane 1) or a FEN1 cDNA starting at M1, M37 or M65 (lanes 2–4, respectively) and a trypsin-treated mitochondrial lysate (Mt) (lane 5).

A standard test of cellular localization is to express tagged proteins in cells and determine their distribution by immunocytochemistry. Therefore, human osteosarcoma (HOS) cells were transfected with Fen1 variants, tagged with hemagluttinin (HA) at their carboxyl-termini. Full-length FEN1 (FEN11.HA) was targeted principally to the nucleus, as per the endogenous protein (Figure 2A). To distinguish dATI from proteolytic processing we substituted the first methionine of FEN1 with isoleucine (FEN1M1I.HA). If the mitochondrial isoform was generated via proteolytic processing of full-length FEN1 then the mutation should result in the loss of both isoforms. However, FEN1M1I.HA yielded a protein that co-localized exclusively with mitochondria (Figure 2B), consistent with a dATI mechanism of synthesis; that is, translation initiates at a site, downstream of M1, generating a mitochondrial isoform of FEN1. In order to determine the internal start codon responsible for mitochondrial targeting, the cellular location of N-terminally truncated FEN1 cDNAs was assessed. FEN137.HA was nuclear-localized (Figure 2C); FEN165.HA, with or without a methionine at position 67 was targeted principally to mitochondria (Figure 2D, S2F), whereas FEN167.HA was predominantly in the nucleus (Figure S2G).

To extend the immunocytochemistry findings, the cellular location of FEN1 was assessed by immunoblotting, after subcellular fractionation. These experiments employed transgenic human cells, expressing tagged full-length FEN1 (FEN11.F) or N-terminal truncated FEN1 (FEN165.F) under a doxycycline-inducible promoter (Figure S3A). Although FEN11.F was more abundant in nuclei, a fraction of it was associated with mitochondria (Figure 2E, S3B). This observation is consistent with published reports of full-length FEN1 in mitochondria [20], despite it lacking a canonical N-terminal MTS. In contrast to FEN11.F, a shorter protein, cross-reacting with the FLAG antibody, was more abundant in mitochondrial extracts than in nuclei (Figure 2E, lanes 2–5), and was assigned as FEN165.F, based on it having the same mobility as the sole FLAG-tagged protein detected in the cell line expressing ectopic FEN165.F (Figures 2F, S3B). The truncated form of FEN1 detected in mitochondria of cells containing full-length cDNA (Figures 2E and S3B) indicates that a single ORF yields two FEN1 variants. Moreover, the size of the endogenous short mitochondrial FEN1 isoform was the same as an untagged FEN1 recombinant protein starting at M65 (Figure 2G, compare lanes 4 and 5). Collectively, these data provided strong support for a mitochondrial isoform of human FEN1 generated via dATI from M65, which we have termed FENMIT, and we revert to calling the full-length protein FEN1. However, the existence of FENMIT left open the questions of its properties and function.

### FENMIT is a R-Loop and a D-loop binding protein

Although FENMIT retains all the residues involved in the primary protein-DNA interaction, it lacks much of the wedge that induces a 100 degree bend in DNA, and several key residues believed to be involved in binding to 5′ and 3′ flaps [27]. Therefore, FENMIT was expected to have different properties to the full-length protein. Specifically, FENMIT was unlikely to be capable of cleaving 5′ single-stranded DNA (ssDNA) flap structures, which are intermediates of Okazaki fragment processing and LP-BER.

**Figure 3:**
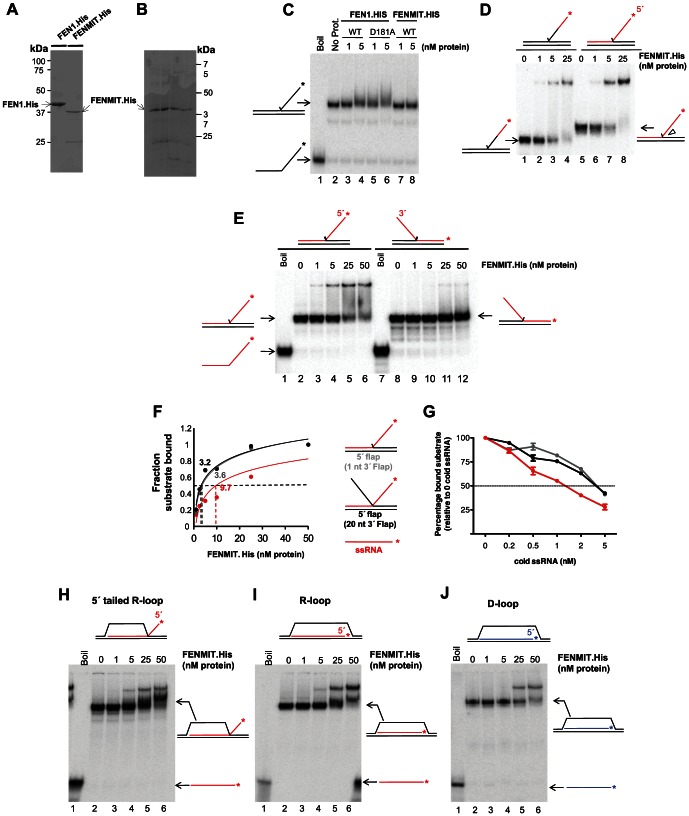
Recombinant FENMIT binds preferentially to RNA flap structures. Recombinant His-tagged FEN1 and FENMIT purified either by (**A**) a single nickel gravity column, or (**B**) HPLC-purified over nickel, DEAE, and SP columns, were fractionated on 4–20% gradient gels, and stained with Coomassie Blue. Prominent bands were excised and analyzed by MALDI-TOF-TOF MS, and all were identified as FEN1, with the exception of an unidentified protein of∼60 kDa unique to (**B**). Gel shift analysis was used to measure recombinant His-tagged FENMIT binding to 0.2 nM of radiolabeled equilibrating 5′ flaps comprising exclusively (**C**) DNA, or (**D**) with 15 nucleotides (nt) of ssRNA at the 5′ end of the flap (T1:U1:19RNA15* (for this and other sequences see Text S1); lanes 1–4) or a full 5′ RNA flap (T2:U2:RNA44*; lanes 5–8). FENMIT binding was analyzed by non-denaturing PAGE. (**E**) Gel shift analysis was used to measure recombinant His-tagged FENMIT binding on 0.2 nM of a radiolabeled equilibrating full 5′ RNA flap (T2:U2:RNA44*; lanes 2–6) and full 3′ RNA flap (T5:U5:RNA44*; lanes 8–12). Schematics of the substrates are indicated above and to the sides of the gel. (**F**) FENMIT (1–50 nM) binding to 0.2 nM of a radiolabelled 5′ flap (with a 1 nt 3′ equilibrating flap) (U2:T2:RNA44*) (gray line), a 5′ flap (with a 20 nt 3′ equilibrating flap) (U3:T2:RNA44*) (black line), or ssRNA (RNA44*) (red line); representative gel images from which the data were derived are shown in Figure S4F. (**G**) Competition assay: the ability of FENMIT (25 nM) to bind to 0.2 nM of the radiolabeled substrates, used in panel C, in the presence of increasing concentrations (0, 0.2, 0.5, 1, 2, and 5 nM) of non-radioactive ssRNA (RNA44); representative gel images from which the data were derived are shown in Figure S4G. Color codes are as panel C. Schematic representation of the substrates used for panels C and D are illustrated between them. Gel shift analysis was used to measure recombinant His-tagged FENMIT binding to 0.2 nM of a radiolabeled (**H**) 5′ tailed R-loop (T7:D2:RNA44*), (**I**) R-loop (T6:D1:RNA44*), and (**J**) D-loop (T7:D1:DNA44*). Schematics of the substrates are indicated above and to the right of the gels.

To evaluate the preferred substrates of FENMIT, wild type (WT) and nuclease-deficient (D181A) forms of FEN1 and FENMIT were purified from bacteria (Figure 3A, 3B). In contrast to FEN1, FENMIT did not cleave the canonical substrate [28], which is an equilibrating double flap with a 5′ ssDNA flap and a 1 nucleotide 3′ flap (Figure S4A and chart). Nor did FENMIT bind to, or cleave, ssDNA, double-stranded DNA (dsDNA), RNA/DNA hybrid (data not shown). While both the wild type and catalytic mutant versions of FEN1 bound to a DNA flap, FENMIT did not bind to this substrate (Figure 3C). However, FENMIT did bind to a mixed RNA-DNA flap substrate, independent of magnesium (Figure 3D, S4B), and the binding efficiency was greatest when the 5′ flap was exclusively RNA (Figure 3D, compare lanes 3 and 4 to lanes 7 and 8). In contrast, FEN1 bound the RNA flap with lower affinity (Figure S4C, compare lanes 3–6 to lanes 7 and 8). We confirmed that recombinant FENMIT forms a complex with the RNA flap structure by inducing a supershift with FEN1 antibodies (Figure S4D). Reversing the structure to create a substrate with a 3′ RNA flap markedly decreased binding (Figure 3E), indicating FENMIT associates specifically with 5′ RNA flaps.

The loss of the first 64 residues of FEN1 removes an acid block loop (residues 56–59) that cannot accommodate a 3′ flap longer than one nucleotide, because of charge repulsion [27]. Hence, extending the 3′ DNA flap to 20 nucleotides inhibited the nuclease action of FEN1 (Figure S4E, lanes 11–14), presumably because it cannot bind to this substrate. In contrast to FEN1, FENMIT bound to RNA-containing 5′ flap structures with short or long 3′ DNA flaps equally well (Figure S4F). Although FENMIT also bound to ssRNA (Figure S4F), its affinity of binding was three-fold lower than that for RNA flap substrates (Figure 3F). Moreover, in competition experiments, radiolabeled RNA flap structures required three times more ‘cold’ ssRNA to compete half of the available FENMIT than was the case for radiolabeled ssRNA (Figure 3G, S4G). The structure with a 5′ RNA flap and an extended 3′ DNA flap resembles an R-loop, and when FENMIT was incubated with a synthetic R-loop, it bound to it with similar affinity (Figure 3H). However, it also bound to an R-loop lacking an RNA flap (Figure 3I), and to a lesser extent, to a D-loop (Figure 3J). Together, these results indicate that dATI-dependent truncation of FEN1 generates a mitochondrial protein with altered substrate recognition and enzymatic properties, converting a protein that recognizes and cleaves flaps, to a protein that binds preferentially to RNA flaps and R-loops.

### FENMIT is recruited to mtDNA under conditions that promote R-loop formation near the origin of replication

**Figure 4:**
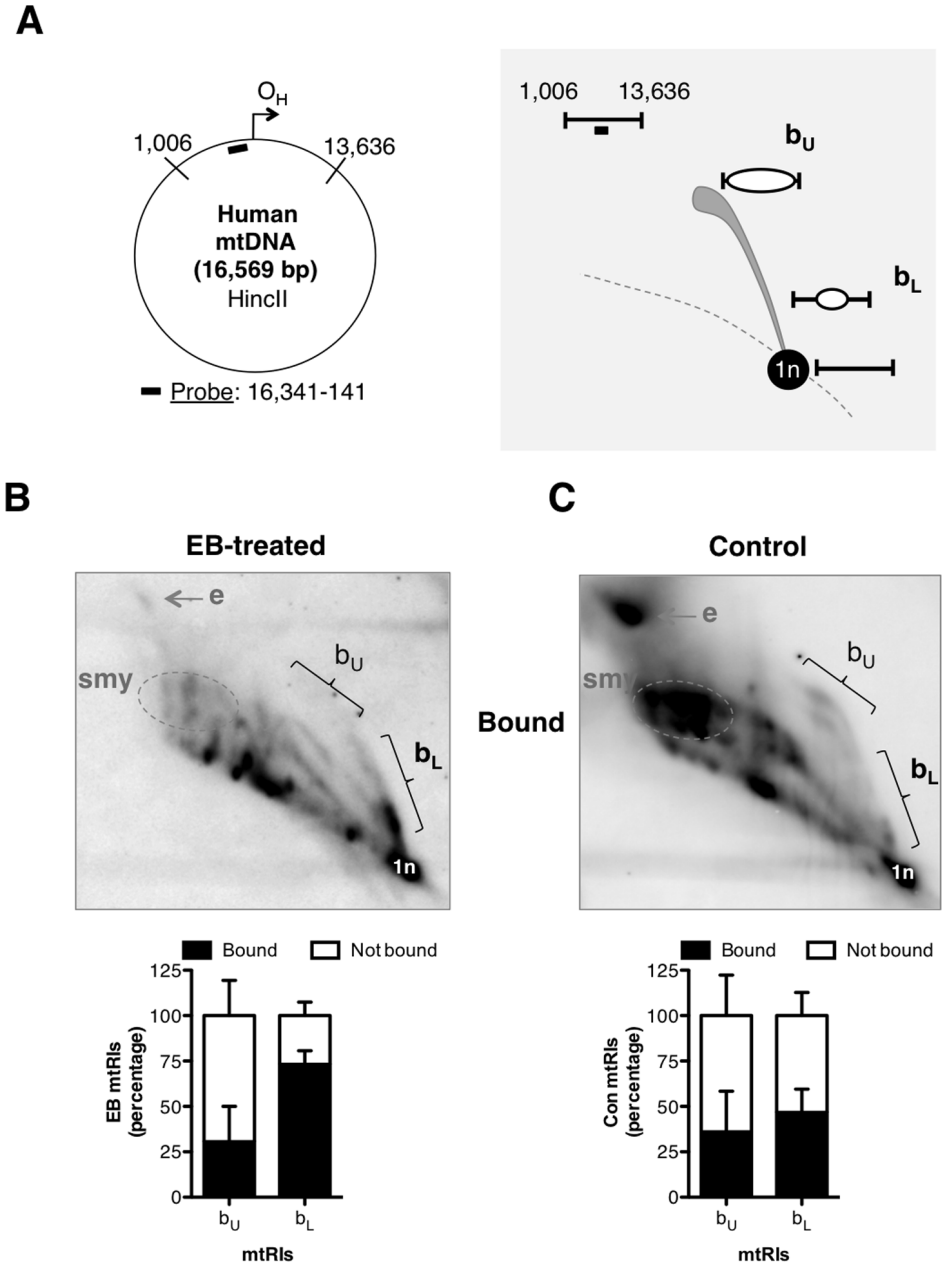
RNA/DNA hybrids accumulate in the origin-containing region after partial mtDNA depletion with EB. (**A**) Schematic map of human mtDNA annotated with HincII restriction sites and a black rectangle to denote the probe. O_H_, origin of heavy strand replication. To the right of the human mtDNA schematic is a cartoon depicting the features of interest in experiments (**B**) and (**C**). 2D-AGE analysis after HincII digestion of mtDNA from sucrose gradient-purified mitochondria, followed by immunocapture using the RNA/DNA hybrid antibody (S9.6) was carried out after: (**B**) 72 h ethidium bromide (EB) treatment (100 ng/ml, 72 h) or (**C**) no treatment (Control). The abundance of the immunocaptured upper (b_u_) and lower (b_L_) portions of the origin-containing bubble arcs were expressed as percentages: per cent bound  =  bound/(bound + unbound) x 100, and are shown below the 2D-AGE images. Small bubble structures that map near the replication origin [34] resolve on the lower portion of the bubble arc (b_L_), and these were more abundant in the EB-treated mtDNA samples. Slow-moving y arcs (smy) and eyebrows (e) are intermediates of RITOLS replication [37], which were drastically reduced in the EB-treated mtDNA samples.

The in vitro data suggested that FENMIT binds to species comprising an RNA that is partially, or fully, hybridized to DNA. Although R-loops formed during transcription pose a threat to genomic integrity, in other contexts R-loops make positive contributions to nucleic acid metabolism [22]. For instance, the initiation of mitochondrial DNA replication is thought to depend on a RNA primer (generated by mitochondrial RNA polymerase, mtRNAP) that is hybridized to the template parental strand, generating an R-loop [29]. Because ethidium bromide (EB) is a potent inhibitor of transcription elongation in mitochondria [30], we inferred that its application would cause RNA synthesis to arrest prematurely, as occurs in vitro [31], resulting in the accumulation of short RNAs. Specifically, the RNA primer at the origin of mtDNA replication, which is two orders of magnitude shorter than the genome-length polycistronic transcripts required for expression of mtDNA, should increase in the presence of EB, creating a substrate for FENMIT. To test this idea, fragments of mtDNA containing RNA/DNA hybrids, were immunocaptured from mitochondria of cells treated with or without EB and analyzed by two-dimensional agarose gel electrophoresis (2D-AGE). Small bubble structures containing regions of RNA/DNA hybrid were more abundant in the EB-treated samples than in controls (Figure 4), suggesting that the chemical causes short R-loops to accumulate on mtDNA in living cells.

**Figure 5:**
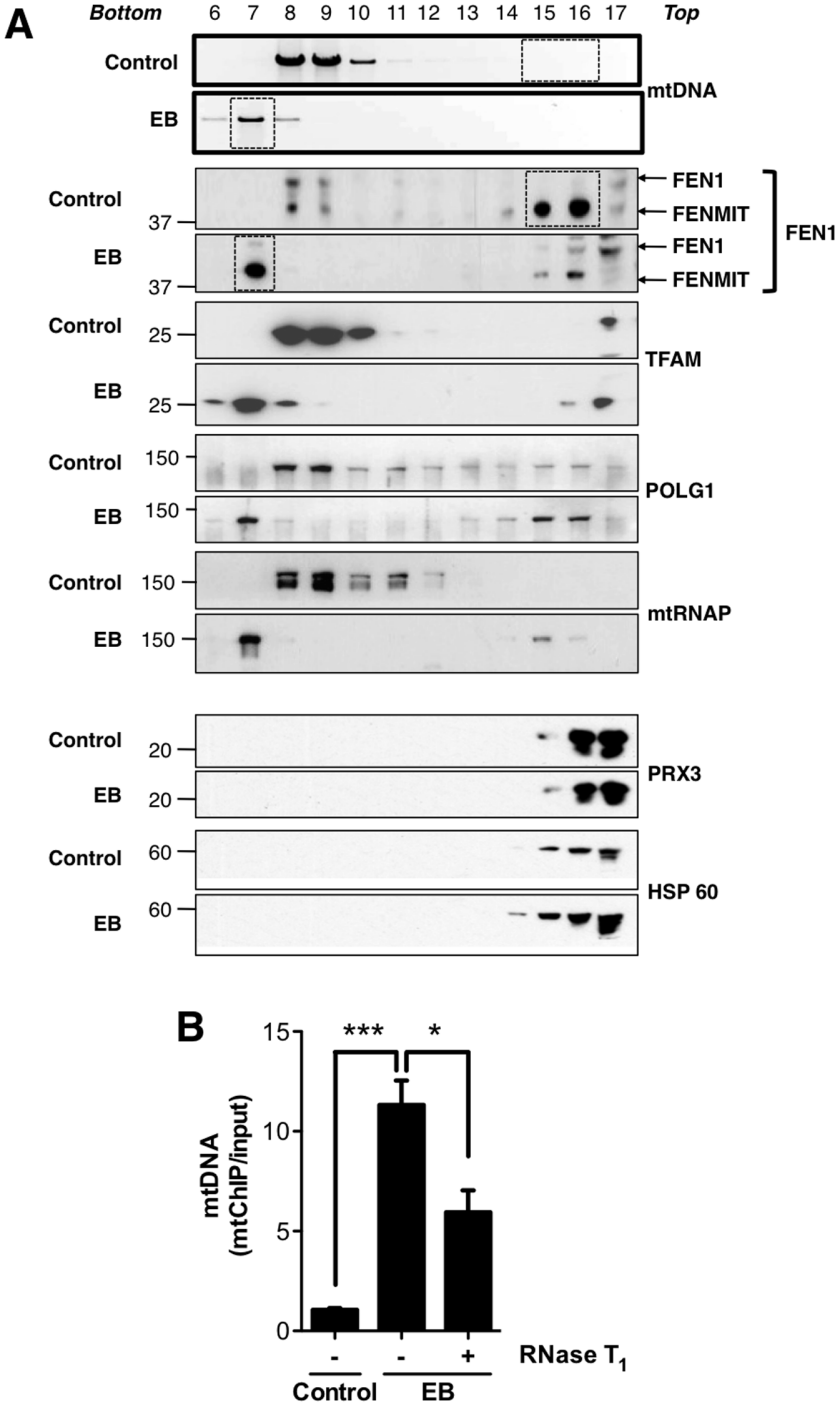
Enhanced, RNA-dependent recruitment of FENMIT to mtDNA in response to EB treatment. Analyses of trypsin-treated mitochondria from control- and ethidium bromide (EB)-treated (100 ng/ml, 72 h) HEK293T cells, after lysis and ultracentrifugation through an Iodixanol gradient[. Fractions, collected from the bottom of the tube are indicated at the top of the figure. Mitochondrial DNA was analyzed via Southern blotting using a probe that spanned nucleotides 16,241–141, according to the human mtDNA reference sequence [49]. FEN1 was detected via immunoblotting. TFAM, POLG1, and mtRNAP are proteins known to bind to mtDNA, whereas PRX3 and HSP60 do not bind to mtDNA. Dashed boxes indicate the submitochondrial location of the majority of FENMIT. (**B**) Mitochondrial lysates from cells, expressing tagged FENMIT, treated without (control) or with EB were incubated with anti-FLAG M2 agarose beads in the absence (-) or presence (+) of RNaseT_1_. Input and elutions (following immunoprecipitation) were spiked with GFP cDNA (550 ng) to provide an internal control for nucleic acid precipitation. Mitochondrial DNA (comparing the abundance of the mtDNA-encoded cytochrome *b* gene (*CYTB*) to that of the nuclear-encoded *APP* gene) and GFP were amplified via qPCR in order to determine FENMIT.F occupancy of mtDNA. Error bars represent standard error of the mean of 3 or 4 separate experiments. ***p<0.01, * p<0.05, using one-way ANOVA.

The use of EB enabled us to investigate the interaction of FENMIT with its predicted substrate in a biological setting. To this end, mitochondria from cells treated with or without EB were sub-fractionated on Iodixanol gradients. Analysis of the gradient fractions by Southern blotting and immunoblotting revealed that the majority of free FENMIT was recruited to mtDNA in the cells treated with EB, despite the marked reduction in mtDNA copy number (Figure 5A, fraction 7 of EB). In contrast to FENMIT, the relative abundance and position of the mtDNA interacting proteins, TFAM, POLG1 and mtRNAP correlated with mtDNA, irrespective of EB exposure (Figure 5A).

Although full-length FEN1 has been linked to DNA repair in mitochondria, the recruitment of FENMIT to mtDNA after prolonged EB treatment cannot be attributed to a DNA damage response, as EB is a poor mutagen of mtDNA [32]. Furthermore, DNA damaging agents in the form of oxidative stress or UV irradiation did not enhance the recruitment of FENMIT to mtDNA (Figure S5).

### FENMIT binding to mtDNA is dependent on RNA in human cells

Because recombinant FENMIT binds to RNA flaps and R-loops (Figure 3) yet associates with mtDNA when transcription is repressed (Figure 5A), it was important to verify that FENMIT was recruited to mtDNA molecules bearing RNA. Therefore, tagged FENMIT was used as bait to co-immunoprecipitate mtDNA from cells treated with or without EB. Under all conditions, FENMIT was enriched by the immunoprecipitation procedure (Figure S6, lanes 7–10). The proportion of mtDNA that co-precipitated with FENMIT was significantly greater (>12-fold) when cells were exposed to EB (Figure 5B), and a small amount of the mtDNA packaging protein, TFAM, was detected exclusively in EB-treated samples (Figure S6, lane 9). This confirms that the movement of endogenous FENMIT on the Iodixanol gradient, in response to EB exposure, is attributable to a physical association with a modified form of mtDNA, or nucleoprotein. Importantly, the use of RNase T1, to remove single-stranded RNA, decreased the amount of mtDNA that co-precipitated with FENMIT by∼55% (Figure 5B), suggesting that binding of FENMIT to mtDNA is at least partly dependent on the presence of RNA.

### Elevated expression of FENMIT inhibits mtDNA replication after EB treatment

**Figure 6:**
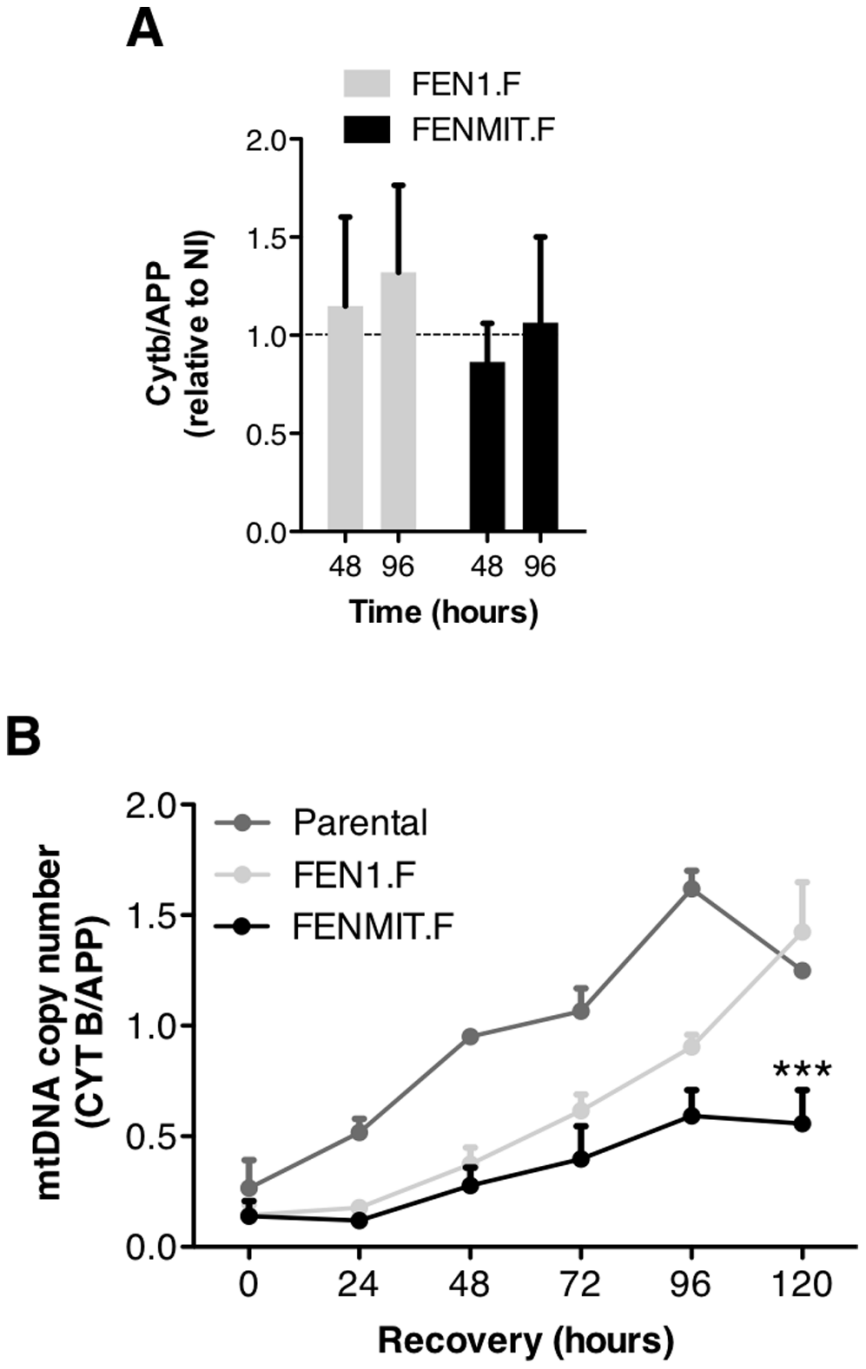
FENMIT overexpression inhibits recovery of mtDNA copy number after transient mtDNA depletion. (**A**) HEK293T cell lines overexpressing FEN1.F or FENMIT.F (by addition of 10 ng/ml doxycycline) for the indicated times (48 h and 96 h). The copy number of mtDNA is expressed relative to non-induced (NI) cell line values set to 1 (dashed line). (**B**) Following partial depletion of mtDNA with EB (100 ng/ml) for 72 h, mtDNA was allowed to recover for 120 h, and samples were taken every 24 h. Mitochondrial DNA copy number was determined by qPCR, comparing the abundance of the mtDNA-encoded cytochrome *b* gene (*CYTB*) to that of the nuclear-encoded *APP* gene. Error bars represent standard error of the mean of 2 or 3 separate experiments. ***p<0.01, using two-way ANOVA.

The RNA-dependent binding of FENMIT to mtDNA, and its recruitment to mtDNA after EB treatment when there is also an increase in R-loops near the origin of replication, suggests FENMIT might influence the initiation of replication in mitochondria. If true, then FENMIT overexpression should be most detrimental during the surge of mtDNA replication that follows transient depletion [33]. Accordingly, whilst FENMIT overexpression had no marked effect on mtDNA copy number under standard culture conditions (Figure 6A), it significantly inhibited the amplification of mtDNA following transient depletion (Figure 6B). Therefore, we infer that an excess of FENMIT stabilizes R-loops at the origin, and the impediment to mtDNA recovery implies that processing of R-loops is necessary for the progression of replication.

## Discussion

The proposed derivative of FEN1, FENMIT, adds to the growing list of mitochondrial isoforms, whose targeting to the organelle depends on alternative translation initiation from an internal start site [23]. Because ATI removes a highly conserved portion of FEN1 that is critical to its canonical function [27], it was inevitable that FENMIT would have different properties to full-length FEN1. The first 64 residues include the hydrophobic wedge that forms part of the track that distorts the path of the template strand, enabling the active site to gain access to the scissile phosphate [27]. Hence, cleavage of flap structures by FENMIT was not detected. Because, FEN1 cleaves RNA flaps, whereas FENMIT stabilizes them, the proteins might have opposing functions. The computational software ‘BindN’ predicts that full-length FEN1 should bind to RNA in preference to DNA (see Text S1). Notwithstanding this, initiation at residue 65, to create FENMIT, removes a portion of the protein that is predicted by BindN to prefer DNA to RNA, and so FENMIT's preference for RNA should be greater than that of FEN1, which is concordant with the findings of this report. In any case, the current study demonstrates that the first 64 residues of FEN1 are key to its nucleic acid binding properties, as without this region RNA is the preferred substrate of the protein. Ultimately a crystal structure of FENMIT in complex with RNA and RNA flap structures will be needed to understand the critical protein-nucleic acid interactions.

FENMIT binds to synthetic RNA-containing structures, resembling 5′ RNA flaps and R-loops (Figure 3), and RNA flaps contribute to the recruitment of FENMIT to DNA, as an enzyme that can trim such flaps (RNase T1) decreased the amount of mtDNA co-immunopurifying with FENMIT (Figure 5B). These nucleic acid binding properties make the protein highly unusual, if not unique, and there are particular features of mtDNA metabolism that could utilize these properties of FENMIT. Notably, R-loops have been implicated in the initiation of mtDNA replication. Transcription by mtRNAP from the light strand promoter (LSP) in the noncoding region (NCR) gives rise to short transcripts, whose 3′ ends are close to prominent 5′ ends of DNA that delineate the origin/terminus of replication, known as OH [29]. R-loops may have a propensity to form in the NCR of mtDNA because of the high GC content downstream of LSP, as elsewhere this is correlated with an increased incidence of R-loop formation [22]. Therefore, we posit that one function of FENMIT is to secure the R-loop between LSP and OH, to facilitate the initiation of mtDNA replication (Figure S7A, S7B). Equally, it might play a role in the initiation of mtDNA replication from other sites in the NCR [34]. This model marries the biochemical properties of FENMIT, the accumulation of early replication intermediates containing RNA/DNA hybrid and FENMIT's RNA-dependent association with mtDNA.

Interestingly, the short RNA from LSP to OH contains a G-rich sequence that can form a G-quadruplex structure in vitro [35]. Thus, RNA synthesis in the OH primer region might produce an interrupted R-loop providing the ideal substrate for FENMIT (Figure S7C). G-quadruplexes notwithstanding, once the transition from RNA to DNA synthesis occurs the structure becomes part R-loop and part D-loop and so the ability of FENMIT to bind to D-loop structures might aid its retention at the origin beyond the initiation of DNA synthesis.

Although RNA is incorporated throughout the lagging strand during mtDNA replication [36], [37], FENMIT does not appear to be tightly associated with mtDNA under standard growth conditions when this mode of replication is active. Nevertheless, FENMIT might facilitate the use of lagging strand RNA for DNA repair. Mitochondria contain reverse transcriptase activity in the form of telomerase [38], and the lagging strand RNA could be used as a template for repairing or bypassing lesions on the leading strand, in a process aided by FENMIT. Finally, FENMIT might play a role in the processing of polycistronic mitochondrial transcripts, as this is believed to occur while the RNA is partly bound to DNA [39]. Because of the unique mechanisms of replication and expression of mtDNA, none of these possible functions is applicable to the nucleus, which would explain why the truncated form of FEN1 is detectable only in the mitochondrial compartment of the cell.

Ablating FENMIT expression while preserving that of FEN1 would help clarify its role in mtDNA metabolism. However, Fen1 is essential in mammals [16] and the key residue for dATI is located in a region of the hydrophobic wedge required for substrate recognition. Hence, designing a fully functional FEN1 whose transcript cannot yield FENMIT via dATI will be challenging. Because of the high degree of sequence conservation of FEN1, it is not clear how many organisms create a dATI-dependent mitochondrial isoform of FEN1, and whether M65 is conserved in order to maintain the function of FEN1 or to generate FENMIT. Thus, whilst M65 is preserved from yeast to human, dATI need not be the driving force behind this evolutionary conservation.

## Materials and Methods

### Cell culture and construct designs

HOS, HeLa, Fibroblasts, and Flp-In™ T-Rex™ 293 (HEK293T) cells (Invitrogen) were cultured in DMEM, 0.1% penicillin/streptomycin, 10% FBS. DG75 cells were cultured in IMDM. Stable HEK293T transformants were selected with Hygromycin B. Mutant constructs were made using the QuikChange site-directed mutagenesis kit (Stratagene).

### Confocal microscopy

HOS cells were transfected using Lipofectamine 2000 with Fen1 cDNAs. 24 hours post transfection, cells were stained with Mitotracker, fixed with paraformaldehyde, and incubated with anti-HA (Roche Diagnostics) antibody. Subsequently, cells were incubated with Alexa Fluor 488 goat anti-rat IgG. Coverslips were mounted with 1,4-diazabicyclo[2.2.2]octane (Sigma), containing 4′,6-diamidino-2-phenylindole, dihydrochloride (DAPI). Images were acquired with an LSM 510 META confocal microscope (Carl Zeiss, Jena, Germany).

### Immunoblotting

Proteins were resolved by 4–12% NuPAGE Bis-Tris SDS-PAGE (Invitrogen) and transferred to nitrocellulose membrane. Immunoblotting was done as described previously [24].

### Mitochondrial Import

[35S]-methionine-labeled proteins were incubated with rat liver mitochondria as previously described [24].

### Nuclear, cytosolic, and mitochondrial isolation from cultured cells

HOS or HEK293T cells were homogenized in hypotonic buffer (20 mM Hepes-NaOH [pH 7.8], 5 mM KCl, 1.5 mM MgCl2, 2 mM DTT, 1 mg/ml BSA, 1 mM PMSF, Protease Inhibitor Cocktail (Roche)). Low speed centrifugation of HEK293T cells resulted in a pellet that was used to isolate intact nuclei. Cytosolic extracts were obtained from post-mitochondrial supernatants, and mitochondria were prepared as described previously [24].

### Quantification of mtDNA copy number

Total DNA was extracted from cell lysed in proteinase K (PK) Buffer (20 mM HEPES, pH 7.8; 75 mM NaCl; 50 mM EDTA; 0.2% SDS; 0.2 mg/ml PK) for 2 hours at 50°C. After precipitation and resuspension in TE buffer, pH 8.0, 25 ng DNA lots of DNA were used for Q-PCR with Amplitaq Gold (Applied Biosystems) according to manufacturer's instructions, except that 5% w/v glycerol was added. Primers and probes are listed in Text S1. Cycle conditions were 95°C for 10 min, followed by 40 cycles of 95°C for 0.25 min and 60°C for 1 min).

### Mitochondrial DNA analysis

mtDNA was extracted and analyzed by one or two dimensional agarose gel electrophoresis and Southern hybridization as previously described [24], [33]. Primers and probes are listed in Text S1.

### Co-immunoprecipitation

Mitochondria were purified from FENMIT.F-expressing HEK cells, lysed, and incubated with FLAG M2 agarose beads (Sigma). RNase T1 buffer (10 mM Tris, pH 7.4; 5 mM EDTA; 300 mM NaCl) was added to mitochondrial lysates from ethidium bromide-treated cells along with 1000 units of RNase T1 (Roche) or no enzyme (control). FENMIT.F was eluted with Flag peptide. Inputs and elutions were spiked with GFP cDNA (550 ng) in order to use as an internal control for nucleic acid precipitation. mtDNA and GFP were amplified via qPCR, as previously described [40], in order to determine FENMIT.F occupancy of mtDNA.

### In silico mitochondrial localization prediction

Methionine residues occurring at the amino terminus of the FEN1 amino acid sequence were queried for possible dATI, with mitochondrial targeting predicted using Mitoprot [41], TargetP [42], Predotar [43], PSORTII [44] and iPSORT [45].

### S9.6 antibody purification

Crude supernatant from S9.6-expressing hybridoma cells [46] was a gift from Dr. Andrew Jackson. The mouse monoclonal S9.6 antibody was purified from the supernatant using a recombinant Protein A/G spin column (Pierce) to a final concentration of 0.7 mg/ml.

### Immunopurification of RNA/DNA hybrids

DNA from HEK cell mitochondria was digested with HincII, precipitated, and resuspended in binding buffer (10 mM HEPES, pH 7.2; 50 mM NaCl; 10 mM EDTA). Immunocapture was as previously described [47].

### FEN1 purification

E. coli (BL-21 strain) were grown to an OD600 of 0.4 to 0.6. FEN1 variants were overexpressed using 5 mM IPTG and cells were grown overnight at 25°C. Cells were sonicated and centrifuged. The supernatant was clarified through a 0.2 µm filter. For single gravity column purification, bacterial cell lysate was run through a Ni Sepharose gravity column in the presence of Imidazole (5 mM). Column was washed with 10 volumes of PN buffer (40 mM NaPO4, pH 7.4; 0.1 M NaCl)+5 mM Imidazole, two column volumes with 60 mM Imidazole, and then eluted with 1 M Imidazole. For HPLC purification, bacterial lysate from cells that had been induced with 1 mM IPTG was run through a Ni Sepharose column, washed with 0.3 M Imidazole and eluted with 1 M Imidazole. The Ni Sepharose elution was loaded onto a DEAE to remove contaminating proteins that might bind nucleic acids, and then sample was loaded on to a HiTrap SP column, eluted with a linear NaCl gradient, and dialyzed against Protein storage buffer (40 mM NaPO4, pH 7.4; 0.1 M NaCl; 50% glycerol), and flash frozen in liquid nitrogen and stored at−80°C.

### Nuclease and Binding assays

All substrates for binding and nuclease assays were gel purified prior to their use and 32P was incorporated at the 5′ end of the oligonucleotides for detection purposes. Recombinant proteins were incubated with 0.2 nM radiolabeled substrates in FEN1 Buffer (25 mM Tris, pH 8.0; 1 mM DTT; 0.1 mg/ml BSA; 6% glycerol). Binding reactions were 1 h on ice, whereas nuclease reactions, with 10 mM MgCl2, were 30 min at 37°C. The reaction products were electrophoresed on 10% non-denaturing polyacrylamide gels in tris-borate buffer and visualized on the Typhoon 9410 Variable Mode Imager (GE Healthcare). Oligonucleotide sequences for FEN1 nuclease and gel-shift analyses are listed in Text S1.

## Supporting Information

Figure S1 **Long and short forms of FEN1 are expressed in several human cell lines, the short form is consistent with translation initiation from an internal methionine and is predicted to be targeted to mitochondria.**(**A**) Expression of full-length FEN1 and a shorter protein (FEN1^S^) were detected by a FEN1 antibody in trypsin-treated human mitochondria of osteosarcoma (HOS) cells, DG75 lymphoblasts and primary fibroblasts (Fibro). Trypsin-treated mitochondria, purified from HOS cells were lysed and centrifuged through an Iodixanol gradient. The migration of mtDNA was detected using a probe that spanned a region within the NCR (nt 16,241–141) according to the human mtDNA reference sequence (49). Endogenous FEN1, TWINKLE and TFAM were evaluated by immunoblotting. (**B**) Separation of HEK293T mitochondrial lysates through an Iodixanol gradient, followed by immunoblotting of FEN1 and TFAM. (**C**) N-terminal amino acid sequence alignment of FEN1 between Human, Orangutan, Mouse, Pig, Cow, Dolphin, Megabat, and *S. cerevisiae*, and *in silico* mitochondrial targeting prediction scores. M1, M37, M65, and M67 above the alignment correspond to methionines 1, 37, 65 and 67 of FEN1, respectively. Green and black rectangles indicate conserved methionine residues. Red color in the heat map indicates a strong probability of mitochondrial localization.
(EPS)

Figure S2 **dATI and mitochondrial targeting occur primarily with FEN1^65^.** (**A**) SDS-PAGE gel of [^35^S]-methionine-labeled FEN1 polypeptide variants generated *in vitro*. “M67”, “M65”, “M37” and “M1” refer to the methionines where translation starts, all of which have an artificial consensus Kozak sequence (GCCACCAUG). “WT M1” indicates that the AUG encoding M1 is preceded by 21 nucleotides of the native FEN1 mRNA. The *in vitro-*synthesized proteins are depicted schematically below the SDS-PAGE gels, and to the right, translation efficiency from M37 (grey bars) and M65/67 (black bars) is depicted graphically, relative to translation from M1 (set to 1). Translation abundance was determined by densitometric quantification of bands corresponding to M1, M37, or M65/67, corrected for background. (**B**) Nucleotides flanking AUG codons encoding M1, M37, M65, and M67 of Fen1 mRNA. Annotated, M1 AUG codon is labeled green and the putative dATI AUG codon is labeled in red. Larger font indicates Kozak sequence conservation with the optimal Kozak sequence: (A/G)CC(A/G)CCAUGG. (**C**) Partial DNA sequence alignment of Fen1 from human to yeast. Yellow bars delineate two out-of-frame sORFs. Clear boxes - two out-of-frame ATG codons that mark the beginning of sORF1 and sORF2, and the M37-ATG codon. Blue box - out-of-frame stop codon (TGA) of the two sORFs. Red box - the M65-ATG codon. (**D**) SDS-PAGE gel of [^35^S]-methionine-labeled FEN1 polypeptide variants generated *in vitro*. WT, wild type; ΔsORF1, ΔsORF2, and ΔsORF1+2 indicate Fen1 cDNAs containing synonymous mutations that ablate sORF1, sORF2, or both sORF1 and 2. The graph to the right shows the quantification of the different proteins generated from each *in vitro* reaction. Dashed line on the graph marks translation events starting from M37 using WT Fen1 mRNA. Error bars represent standard error of three independent experiments. *p<0.05 using one-way ANOVA. (**E**) Import of [^35^S]-methionine-labeled TFAM (positive control) and FEN1 variants into rat liver mitochondria. 1 µM FCCP (lanes 5–7) was used to dissipate membrane potential. White arrowhead indicates the most prominent FEN1 form that is imported into mitochondria. To the right, import efficiency was determined by densitometric quantification of the trypsin-resistant band (lane 3), relative to total protein surviving incubation with mitochondria (lane 2). Quantification was expressed relative to TFAM import, which was arbitrarily set as 100%. Start methionines are indicated to the right of the gel images. Error bars represent standard error of the mean (n = 3 or 4 experiments). ***p<0.001 using one-way ANOVA. Immunocytochemistry of HOS cells transiently transfected with human FEN1 constructs with HA-tags on the C-terminus starting translation at (**F**) M65, and containing a methionine to isoleucine point mutation at residue 67 (FEN1^65/M67I^.HA), and starting translation at (**G**) M67 (FEN1^67^.HA). Recombinant proteins were labeled with anti-HA antibody (green), while nuclei (blue) and mitochondria (red) were visualized by staining cells with DAPI and Mitotracker, respectively.
(EPS)

Figure S3 **The truncated isoform of FEN1 corresponds in size to a product initiating translation from M65 in human cells.** (**A**) Immunoblotting of inducible HEK293T cells after selection of clones expressing FLAG-tagged full-length FEN1 (FEN1^1^.F) and truncated FEN1 forced to start translation from M65 (FEN1^65^.F). Transgene expression was induced with 1 or 3 ng/ml doxycycline (Sigma) for 24 h. (**B**) Nuclei were run beside purified mitochondria isolated from HEK293T cells expressing FEN1^1^.F or FEN1^65^.F transgenes (1 ng/ml doxycycline for 24 h). Mitochondria were subjected to trypsin protection assays, prior to lysis and immunoblotting. Nuclei (lanes 1, 2, 8, and 9), black slope indicates increasing trypsin concentrations of 0 µg/ml (lanes 3 and 10), 10 µg/ml (lanes 4 and 11), 50 µg/ml (lanes 5 and 12), 100 µg/ml (lanes 6 and 13), and samples treated with both 1% Triton-X100 and 100 µg/ml trypsin (lanes 7 and 14). Ectopic FEN1 was detected with anti-FLAG. GAPDH was used as a loading control in A.
(EPS)

Figure S4 **Endonuclease and nucleic acid binding properties of recombinant human FEN1 variants.** Recombinant FEN1 variants (1 and 5 nM) and 0.2 nM of radiolabeled (**A**) equilibrating 19 nt 5′ ssDNA flap substrate (T1:U1:19DNA*). FEN1 cleavage was assessed by non-denaturing 10% PAGE, with the cleavage products formed by FEN1.His in panel C arbitrarily set as 100%, and graphically represented to the right of the gel image. (**B**) Substrates with an equilibrating 5′ flap containing 15 nt of ssRNA at the 5′ end of the flap (T1:U1:19RNA15*) were incubated with recombinant FEN1 variants in the presence of MgCl_2_ (10 mM) at 37°C for 30 min. Schematics of the substrates and products are indicated to the left of the gel images. (**C**) Gel shift analysis was used to measure binding of 1 nM and 5 nM of recombinant WT FEN1^1^.His (lane 3 and 4), D181A mutant (lane 5 and 6), and FENMIT.His (lane 7 and 8) to 0.2 nM of a radiolabeled 5′ RNA flap (with a 1 nt 3′ equilibrating flap) (U2:T2:RNA44*). FENMIT was purified by two separate methods, indicated above the gel images. (**D**) Recombinant FENMIT was incubated with radioactive substrate and 5 mg of isotype IgG, FEN1 (Genetex), or FEN1 (Bethyl) antibodies for 1 h on ice and analyzed by non-denaturing 10% PAGE. (**E**) Nuclease activity of FEN1.His was assessed by incubation with T1:U1:19DNA* (lanes 3–6), U2:T2:RNA44* (lanes 7–10), and a 5′ flap with a 20 nt 3′ equilibrating flap (U3:T2:RNA44*, lanes 11–14). DNA and RNA (lanes 1 and 2) refer to single-stranded, radiolabeled oligos released from flap substrates after boiling. Substrate and cleavage products are indicated with arrows. (**F**) Gel shift analysis was used to measure binding of FENMIT (1–50 nM) to 0.2 nM of a radiolabeled ssRNA (RNA44*, lanes 1–4 and 13–16), U2:T2:RNA44* (lanes 5–8 and 17–20), and U3:T2:RNA44* (lanes 9–12 and 21–24). Protein concentrations, and schematic representation of substrates used are indicated above the gel images. Combined data from two independent experiments appear in Fig. 3F. (**G**) The ability of increasing concentrations (0, 0.2, 0.5, 1, 2, and 5 nM) of non-radioactive (cold) ssRNA (RNA44) to compete with 0.2 nM radiolabeled RNA44, U2:T2:RNA44, or U3:T2:RNA44 for binding of 25 nM FENMIT.His. Combined data from two independent experiments appear in Fig. 3G.
(EPS)

Figure S5 **The majority of FENMIT remains free of mtDNA in the presence of oxidative stress and DNA damaging agents.** (**A**) Cells were treated with 200 µM of H_2_O_2_ for 1 h followed by mitochondrial extraction or (**B**) exposed to 20 Joules/m^2^ of UV and then allowed to recover for 18 h prior to mitochondrial extraction. Mitochondrial lysates were centrifuged through Iodixanol gradients. Fractions were collected from the base of the gradient tube and are indicated at the top of the figure (5–18). Proteins were detected by immunoblotting. TFAM was used to detect the migration of a known mtDNA-binding protein.
(EPS)

Figure S6 **Immunoblotting following immunoprecipitation of FENMIT.F from mitochondria of cells treated with and without EB.** Immunoblots detecting transgenic FENMIT.F, and endogenous proteins (TFAM and mtRNAP) after immunoprecipitation with Anti-Flag® M2 Affinity gel (Sigma) from purified mitochondrial lysates. Control and ethidium bromide (EB) samples were also treated with (+) or without (-) RNase T_1_ (1000 Units) during FLAG immunoprecipitation.
(EPS)

Figure S7 **Model of FENMIT binding to mitochondrial DNA at the origin of replication.** The mitochondrial RNA Polymerase (mtRNAP) transcribes an RNA (red line) molecule from the light strand promoter (LSP) that can serve as a primer for H-strand DNA (blue line) synthesis by DNA polymerase γ (POLG). The RNA primer must minimally be in the form of an RNA/DNA hybrid at the RNA-DNA transition point, and so there is expected to be an R-loop in this region, to which FENMIT can bind (**A**). Partial dissociation of the RNA/DNA hybrid to produce a RNA tail (**B**), or the formation of a G-quadruplex (**C**) (35) would further facilitating FENMIT recruitment in the vicinity of the H-strand DNA initiation site. Hence, FENMIT might bind to and stabilize the primer RNA to regulate the initiation of mtDNA replication.
(TIF)

Text S1 This file includes supplementary materials and methods and the in silico predicted DNA and RNA binding properties of FEN1 and FENMIT.
(DOCX)
