# Supplementary figures and images for: A Cryptic Targeting Signal Creates a Mitochondrial FEN1 Isoform with Tailed R-Loop Binding Properties
Source: PLoS One. 2013 May 13;8(5):e62340. doi: 10.1371/journal.pone.0062340 (PMC3652857; doi:10.1371/journal.pone.0062340)

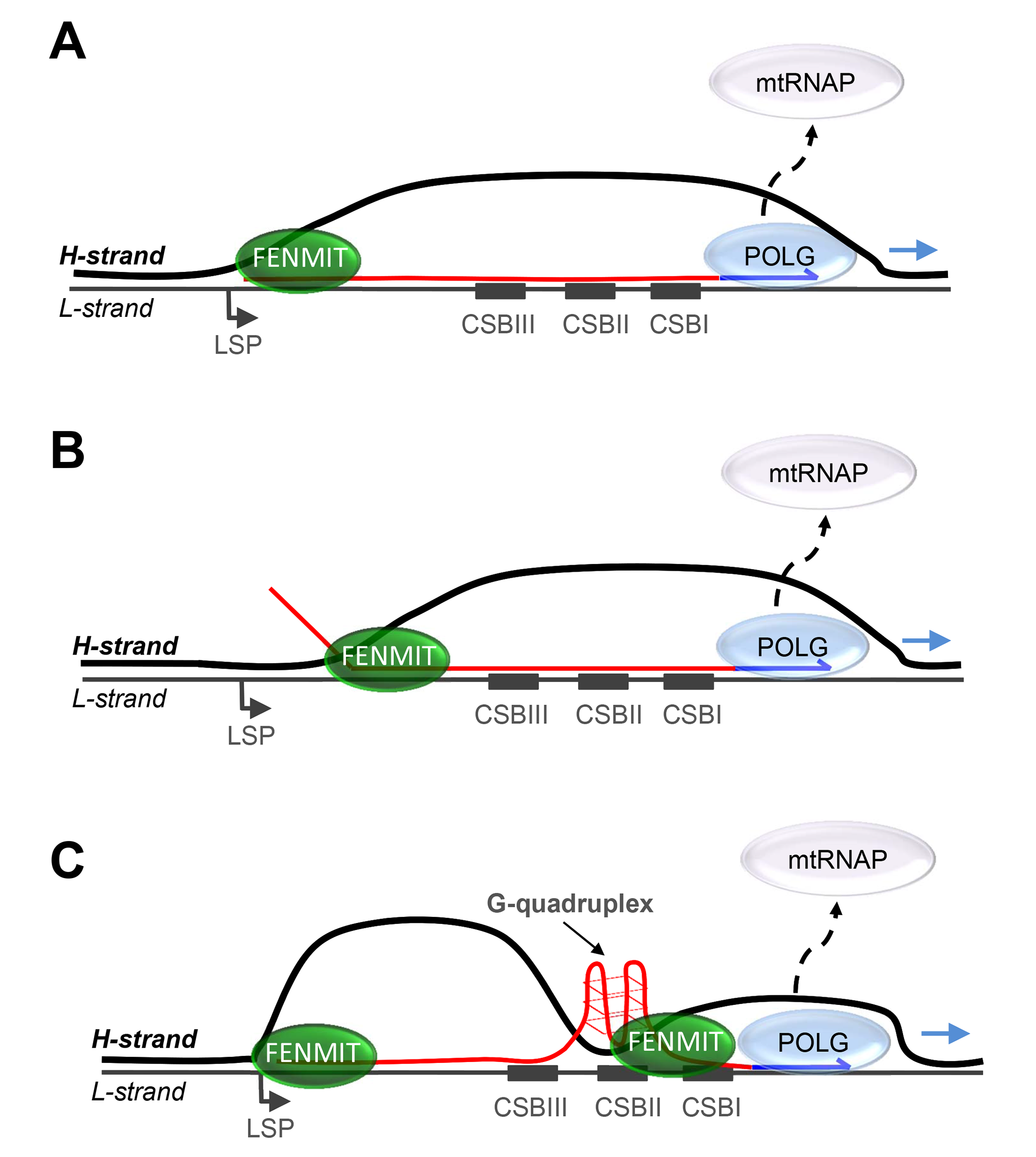

Supplement: Figure S7 — Model of FENMIT binding to mitochondrial DNA at the origin of replication. The mitochondrial RNA Polymerase (mtRNAP) transcribes an RNA (red line) molecule from the light strand promoter (LSP) that can serve as a primer for H-strand DNA (blue line) synthesis by DNA polymerase γ (POLG). The RNA primer must minimally be in the form of an RNA/DNA hybrid at the RNA-DNA transition point, and so there is expected to be an R-loop in this region, to which FENMIT can bind (A). Partial dissociation of the RNA/DNA hybrid to produce a RNA tail (B), or the formation of a G-quadruplex (C) (35) would further facilitating FENMIT recruitment in the vicinity of the H-strand DNA initiation site. Hence, FENMIT might bind to and stabilize the primer RNA to regulate the initiation of mtDNA replication. (TIF) [file pone.0062340.s007.tif]
