# Supplementary material for: A Cryptic Targeting Signal Creates a Mitochondrial FEN1 Isoform with Tailed R-Loop Binding Properties
Source: PLoS One. 2013 May 13;8(5):e62340. doi: 10.1371/journal.pone.0062340 (PMC3652857; doi:10.1371/journal.pone.0062340)
Supplement: Text S1 — This file includes supplementary materials and methods and the in silico predicted DNA and RNA binding properties of FEN1 and FENMIT. (DOCX) [file pone.0062340.s008.docx]

**SUPPORTING INFORMATION**

**Material and Methods**

**Oligonucleotides for FEN1 nuclease and gel-shift analyses**. Oligonucleotide primers are indicated 5′ to 3′. **T1**: GTCGACCTGCAGCCCAAGCTTGCGTTGCTG; **U1:** CAGCAACGCAAGCTTGC; **19DNA:** ATGTGGAAAATCTCTAGCAGGCTGC AGGTCGAC; **19RNA15:** AUGUGGAAAAUCUCUAGCAGGCTGCAGGTCGAC; **T2:** GCGATTATCCGTATCGGACGTAGGAAGCCTGCCATTTGATGGTC; **U2:** GACCATCAAATGGCAGGCTG ; **RNA44:** UCAACGUGGGCAAAGAAUGUCCUACG UCCGAUACGGAUAAUCGC; **T5:** AGCCTGCCATTTGATGGTCGTAGGACATTC TTTGCCCACGTTGA; **U5:** AGACCATCAAATGGCAGGCT; **U3:** GACCATCAA ATGGCAGGCTGAACGAACAAAGCGAAAGCG; **D1:** CCGGTTAGGCTATCTACCC ATAAGTTTATTCCCTGCTCTTAGCAGATGGAGTCTGTGAACTGCAATCAATACCA TCTTAAGCCGACGCAG; **T6:** CTGCGTCGGCTTAAGATGGTGCGATTATCCGTAT CGGACGTAGGACATTCTTTGCCCACGTTGATGGGTAGATAGCCTAACCGG; **DNA44:** TCAACGTGGGCAAAGAATGTCCTACGTCCGATACGGAT AATCGC; **D2:** CCGGTTAGGCTATCTACCCATCGGCTAGGACACGGTGTCTAAGCTATGTAATA G CCGTTGCCGAATATAAACCATCTTAAGCCGACGCAG; **T7:** CTGCGTCGGCTTAA GATGGTGCGATTATCCGTATCGGACGTAGGAGACACCGTGTCCTAGCCGATGGGTAGATAGCCTAACCGG.

**Oligonucleotides for quantification of mtDNA copy number.** Sigma Genosys) are listed 5′ to 3′ and were as follows: CYTB forward: GCCTGCCTGATCCTCCAAAT; CYTB reverse: AAGGTAGCGGATGATTCAGCC; CYTB probe: CACCAGACG CCTCAACCGCCTT (5′-TET; 3′-TAMRA); APP forward TTTTTGTGTGCTC TCCCAGGTCT; APP reverse: TGGTCACTGGTTGGTTGGC; APP probe: CCCTGAACTGCAGATCACCAATGTGGTAG (5′-FAM; 3′-TAMRA). For primers and probes to GFP see ref 40.

**Probes for mtDNA and RNA analysis.** Probe for mtDNA was generated via PCR of human genomic DNA using the following primers: sense: 5′-TTACAGTCAAAT CCCTTCTCGT-3′ and antisense: 5′-GGATGAGGCAGGAATCAAAGACAG-3′. Probe for 18SrDNA was generated via PCR of human genomic DNA using the following primers: sense: 5′-GTTGGTGGAGCGATTTGTCT-3′ and antisense: 5′-GGCCTCACTAAACCATCCAA- 3′. Oligo sequences to generate probes for RNA analysis of Fen1 mRNA: sense: 5'-ATGTGCTGCAGAATGAGGAG-3' and antisense: 5'-AAGGTGAGGCAGTCCATGTC-3' and Gapdh mRNA, sense: 5'-ACCA CAGTCCATGCCATCAC-3'; antisense, 5'-TGACAAAGTGGTCGTTGAGG-3'.

***In silico* predicted DNA and RNA binding properties of FEN1 and FENMIT.**

The analysis was based on BindN (<http://bioinfo.ggc.org/bindn/>). Residues predicted to contribute to binding to DNA or RNA were assessed for all the amino acids of FEN1 (1-380) and FENMIT (65-380), and for the segment missing from FENMIT (1-64). Each residue is assigned a confidence score.

|  | **DNA**  Binding residues | **DNA**  Combined confidence scores | **RNA**  Binding residues | **RNA**  Combined confidence scores |
| --- | --- | --- | --- | --- |
| FEN1 | 79 | 521 | 96 | 631 |
| FENMIT | 68 | 467 | 88 | 588 |
| 1-64 | 11 | 54 | 8 | 43 |

The theoretical gain of RNA over DNA binding for FENMIT, based on the confidence scores is 27% with respect to FEN1, and the loss of residues 1-64 predicts an equal reduction in DNA binding for FENMIT. Thus, these differences may contribute to for the observed gain in RNA-binding capacity of FENMIT in vitro, although the arrangement of the residues in its tertiary structure is likely to be as, if not more, important.
